# Supplementary material for: Past changes in and present status of the coastal carbon cycle
Source: Camb Prism Coast Futur. 2023 Aug 7;1:e34. doi: 10.1017/cft.2023.20 (PMC12337590; doi:10.1017/cft.2023.20)
Supplement: Supplementary file 1 [file S2754720523000203sup001.docx]

Table S1 Spatial extent of ecosystems and urban areas with reference (R) and comments (C). In contrast to Table S2, Table S1 includes overlaps. For instance, ‘ocean’ comprises the shelves and the all other submerged coastal ecosystems, and soil includes peat soils and all non-submerged coastal ecosystems.

|  | Ecosystem | Area | | | | | | |
| --- | --- | --- | --- | --- | --- | --- | --- | --- |
|  |  | from | | | to | | | mean |
|  |  | [10^12^ m^2^] | *R* | *C* | [10^12^ m^2^] | *R* | *C* | [10^12^ m^2^] |
| 1 | Salt marshes | 0.02 | ^1^ |  | 0.06 | ^2,3^ |  | 0.04 |
| 2 | Mangroves | 0.08 | ^4^ |  | 0.15 | ^5^ |  | 0.12 |
| 3 | Seagrass | 0.21 | ^6^ |  | 0.60 | ^7^ |  | 0.41 |
| 4 | Macroalgae | 1.40 | ^8^ |  | 6.80 | ^9^ |  | 4.10 |
| 5 | Tidal flats | 0.12 | ^10^ |  | 0.13 | ^10^ |  | 0.13 |
| 6 | Coral reefs (warm water) | 0.25 | ^11^ |  | 0.85 | ^12^ | ^[[1]](#footnote-1)^ | 0.55 |
| 7 | Coral reefs (cold water) | 0.28 | ^13^ |  | 0.28 | ^13^ |  | 0.28 |
| 8 | Estuaries | 1.05 | ^14^ |  | 3.72 | ^15^ |  | 2.39 |
| 9 | Shelves | 26.39 | ^16^ |  | 32.24 | ^17^ |  | 29.31 |
| 10 | Ocean | 361.88 | ^17^ |  | 361.88 | ^17^ |  | 361.88 |
| 11 | Peat (high latitudes) | 3.10 | ^18^ |  | 3.32 | ^19^ |  | 3.21 |
| 12 | Peat (tropics) | 0.60 | ^18^ |  | 0.84 | ^19^ |  | 0.72 |
| 13 | Soils | 125.80 | ^20^ |  | 125.80 | ^20^ |  | 125.80 |
| 14 | Inland waters | 2.79 | ^21^ |  | 4.09 | ^22^ |  | 3.44 |
| 15 | Reservoirs | 0.25 | ^21^ |  | 0.50 | ^22^ |  | 0.38 |
| 16 | Ice cover (land) | 17.93 | ^23^ |  | 18.68 | ^24^ |  | 18.31 |
| 17 | Urban areas | 0.31 | ^25^ |  | 0.73 | ^25^ |  | 0.52 |

Table S2 Spatial extent of ecosystems and urban areas with reference (R, S1 = see Table S1), comments (C) and without overlaps.

|  | Ecosystem | Area | | | | | | |
| --- | --- | --- | --- | --- | --- | --- | --- | --- |
|  |  | from | | | to | | | mean |
|  |  | [10^12^ m^2^] | *R* | *C* | [10^12^ m^2^] | *R* | *C* | [10^12^ m^2^] |
| 1 | Salt marshes | 0.02 | S1 |  | 0.06 | S1 |  | 0.04 |
| 2 | Mangroves | 0.08 | S1 |  | 0.15 | S1 |  | 0.12 |
| 3 | Seagrass | 0.21 | S1 |  | 0.60 | S1 |  | 0.41 |
| 4 | Macroalgae | 1.40 | S1 |  | 6.80 | S1 |  | 4.10 |
| 5 | Tidal flats | 0.12 | S1 |  | 0.13 | S1 |  | 0.13 |
| 6 | Coral reefs (warm water) | 0.25 | S1 |  | 0.85 | S1 |  | 0.55 |
| 7 | Coral reefs (cold water) | 0.28 | S1 |  | 0.28 | S1 |  | 0.28 |
| 8 | Estuaries | 1.05 | S1 |  | 3.72 | S1 |  | 2.39 |
| 9 | Open shelves^[[2]](#footnote-2)^ | 20.13 |  |  | 23.33 |  |  | 21.73 |
| 10 | Open ocean^[[3]](#footnote-3)^ | 329.64 |  |  | 335.50 |  |  | 332.65 |
| 11 | Peat (high latitudes) | 3.10 | S1 |  | 3.32 | S1 |  | 3.21 |
| 12 | Peat (tropics) | 0.60 | S1 |  | 0.84 | S1 |  | 0.72 |
| 13 | Inland mineral soils^[[4]](#footnote-4)^ | 121.30 |  |  | 121.88 |  |  | 121.59 |
| 14 | Inland waters | 2.79 | S1 |  | 4.09 | S1 |  | 3.44 |
| 15 | Ice cover (land) | 17.93 | S1 |  | 18.68 | S1 |  | 18.31 |
| 16 | Urban areas | 0.31 | S1 |  | 0.73 | S1 |  | 0.52 |
|  |  |  |  |  |  |  |  |  |
| 17 | Marine coastal ecosystems^[[5]](#footnote-5)^ | 23.27 |  |  | 35.64 |  |  | 29.46 |
| 18 | Open ocean^[[6]](#footnote-6)^ | 329.64 |  |  | 335.50 |  |  | 332.57 |
| 19 | Land^[[7]](#footnote-7)^ | 146.02 |  |  | 149.54 |  |  | 147.78 |
|  | Total^[[8]](#footnote-8)^ | 499.93 |  |  | 520.68 |  |  | 509.81 |

Table S3 POC burial rates with reference (R) and comments (C). In case no data have been found POC burial was set to zero.

|  | Ecosystem | POC burial | | | | | | | | |
| --- | --- | --- | --- | --- | --- | --- | --- | --- | --- | --- |
|  |  | from | | | to | | | mean | mean^[[9]](#footnote-9)^ | ± range^[[10]](#footnote-10)^ |
|  |  | [g C m^-2^ yr^-1^] | *R* | *C* | [g C m^-2^ yr^-1^] | *R* | *C* | [g C m^-2^ yr^-1^] | Tg C yr^-1^ | |
| 1 | Salt marshes | 73.15 | ^1^ |  | 440.00 | ^26^ |  | 256.57 | 12.82 | 11.36 |
| 2 | Mangroves | 39.94 | ^1^ |  | 229.00 | ^26^ |  | 134.47 | 19.08 | 15.81 |
| 3 | Seagrass | 62.65 | ^1^ |  | 176.00 | ^27^ |  | 119.32 | 59.49 | 46.11 |
| 4 | Macroalgae | 0.36 | ^1^ |  | 3.24 | ^1^ |  | 1.80 | 11.25 | 10.75 |
| 5 | Tidal flats | 99.90 | ^28^ |  | 159.70 | ^28^ |  | 129.80 | 16.73 | 4.32 |
| 6 | Coral reefs (warm water) | 0.11 | ^29^ | ^[[11]](#footnote-11)^ | 1.28 | ^29^ |  | 0.70 | 0.56 | 0.53 |
| 7 | Coral reefs (cold water) | < 0.01 | ^30^ | ^[[12]](#footnote-12)^ | 0.05 | ^30^ |  | 0.03 | 0.01 | 0.01 |
| 8 | Estuaries | 13.44 | ^31^ |  | 47.62 | ^31^ |  | 30.53 | 50.0 | 0.00 |
| 9 | Open shelves | 4.47 | ^32^ |  | 8.57 | ^31^ |  | 6.52 | 145.00 | 55.00 |
| 10 | Open ocean | 0.05 | ^33^ |  | 0.12 | ^34^ |  | 0.09 | 28.55 | 11.45 |
| 11 | Peat (high latitudes) | 18.64 | ^35^ |  | 24.00 | ^36^ |  | 21.32 | 68.72 | 10.96 |
| 12 | Peat (tropics) | 12.80 | ^36^ |  | 100.00 | ^37^ |  | 56.40 | 46.01 | 38.35 |
| 13 | Inland mineral soils | 0.41 | ^38^ |  | 0.41 | ^38^ |  | 0.41 | 50.00 | 0.00 |
| 14 | Inland waters | 14.35 | ^38^ |  | 14.65 | ^39^ |  | 14.50 | 50.00 | 10.00 |
| 15 | Ice cover (land) | 0.00 |  |  | 0.00 |  |  | 0.00 | 0.00 | 0.00 |
| 16 | Urban areas | 0.00 |  |  | 0.00 |  |  | 0.00 | 0.00 | 0.00 |

Table S4 PIC burial rates with reference (R) and comments (C). In case no data have been found POC burial was set to zero.

|  | Ecosystem | PIC burial | | | | | | | | |
| --- | --- | --- | --- | --- | --- | --- | --- | --- | --- | --- |
|  |  | From | | | to | | | mean | mean | ± range |
|  |  | [g C m^-2^ yr^-1^] | *R* | *C* | [g C m^-2^ yr^-1^] | *R* | *C* | [g C m^-2^ yr^-1^] | Tg C yr^-1^ | |
| 1 | Salt marshes | 26.00 | ^40^ |  | 54.00 | ^40^ |  | 40.00 | 1.74 | 1.22 |
| 2 | Mangroves | 0.00 | ^40^ | ^[[13]](#footnote-13)^ | 240.00 | ^40^ |  | 120.00 | 18.28 | 18.28 |
| 3 | Seagrass | 95.29 | ^41^ |  | 157.31 | ^41^ |  | 126.30 | 57.36 | 37.02 |
| 4 | Macroalgae | 0.00 |  |  | 0.00 |  |  | 0.00 | 0.00 | 0.00 |
| 5 | Tidal flats | 0.00 |  |  | 0.00 |  |  | 0.00 | 0.00 | 0.00 |
| 6 | Coral reefs (warm water) | 65.75 | ^42^ |  | 140.00 | ^43^ |  | 102.88 | 67.69 | 51.27 |
| 7 | Coral reefs (cold water) | 0.66 | ^30^ | ^[[14]](#footnote-14)^ | 5.60 | ^30^ |  | 3.13 | 0.89 | 0.70 |
| 8 | Estuaries | 0.00 | ^31^ |  | 0.00 | ^31^ |  | 0.00 | 0.00 | 0.00 |
| 9 | Open shelves | 7.45 | ^31^ |  | 11.32 | ^44^ |  | 9.38 | 207 | 57.00 |
| 10 | Open ocean | 0.40 | ^44,45^ |  | 0.86 | ^46^ |  | 0.63 | 210.18 | 78.07 |
| 11 | Peat (high latitudes) | 0.00 |  |  | 0.00 |  |  | 0.00 | 0.00 | 0.00 |
| 12 | Peat (tropics) | 0.00 |  |  | 0.00 |  |  | 0.00 | 0.00 | 0.00 |
| 13 | Inland mineral soils | 0.00 |  |  | 0.00 |  |  | 0.00 | 0.00 | 0.00 |
| 14 | Inland waters | 0.00 |  |  | 0.00 |  |  | 0.00 | 0.00 | 0.00 |
| 15 | Ice cover (land) | 0.00 |  |  | 0.00 |  |  | 0.00 | 0.00 | 0.00 |
| 16 | Urban areas^[[15]](#footnote-15)^ | 0.00 |  |  | 0.00 |  |  | 0.00 | 0.00 | 0.00 |

**References**

1 Duarte, C. M. Reviews and syntheses: Hidden forests, the role of vegetated coastal habitats in the ocean carbon budget. *Biogeosciences* **14**, 301-310, doi:10.5194/bg-14-301-2017 (2017).

2 Davidson, N. C. & Finlayson, C. M. Extent, regional distribution and changes in area of different classes of wetland. *Marine and Freshwater Research* **69**, 1525-1533 (2018).

3 McOwen, C. J. *et al.* A global map of saltmarshes. *Biodiversity Data Journal* **5**, e11764 (2017).

4 Hamilton, S. E. & Casey, D. Creation of a high spatio-temporal resolution global database of continuous mangrove forest cover for the 21st century (CGMFC-21). *Global Ecology and Biogeography* **25**, 729-738, doi:10.1111/geb.12449 (2016).

5 Spalding, M., Kainuma, M. & Collins, L. *World atlas of mangroves*. 319 (Earthscan Ltf, Dunstan House, London UK, 2010).

6 McKenzie, L. J. *et al.* The global distribution of seagrass meadows. *Environmental Research Letters* **15**, 074041, doi:10.1088/1748-9326/ab7d06 (2020).

7 McLeod, E. *et al.* A blueprint for blue carbon: toward an improved understanding of the role of vegetated coastal habitats in sequestering CO2. *Frontiers in Ecology and the Environment* **9**, 552-560, doi:10.1890/110004 (2011).

8 Krause-Jensen, D. & Duarte, C. M. Substantial role of macroalgae in marine carbon sequestration. *Nature Geoscience* **9**, 737-742, doi:10.1038/ngeo2790 (2016).

9 Charpy-Roubaud, C. & Sournia, A. The comparative estimation of phytoplanktonic, microphytobenthic and macrophytobenthic primary production in the oceans. *Marine Microbial Food Webs* **4**, 31-57 (1990).

10 Murray, N. J. *et al.* The global distribution and trajectory of tidal flats. *Nature* **565**, 222-225, doi:10.1038/s41586-018-0805-8 (2019).

11 Burke, L., Reytar, K., Spalding, M. & Perry, A. Reef at Risk, revisited. 130 (World Resource Institute, Washington, DC, USA, 2011).

12 Wilkinson, C. *et al.* in *The First Global Integrated Marine Assessment: World Ocean Assessment I* (ed Nations United) 817-838 (Cambridge University Press, 2017).

13 Freiwald, A., Fossa, J. H., Grehan, A., J., Koslow, J. A. & Roberts, M., J. . Cold-water coral reefs : out of sight, no longer out of mind. 86 (UNEP World Conservation Monitoring Centre, Cambridge, UK, 2004).

14 Cai, W.-J. Estuarine and Coastal Ocean Carbon Paradox: CO2 Sinks or Sites of Terrestrial Carbon Incineration? *Annual Review of Marine Science* **3**, 123-145, doi:10.1146/annurev-marine-120709-142723 (2011).

15 Kang, Y. *et al.* Areas of the global major river plumes. *Acta Oceanologica Sinica* **32**, 79-88, doi:10.1007/s13131-013-0269-5 (2013).

16 Laruelle, G. G. *et al.* Global multi-scale segmentation of continental and coastal waters from the watersheds to the continental margins. *Hydrol. Earth Syst. Sci.* **17**, 2029-2051, doi:10.5194/hess-17-2029-2013 (2013).

17 Harris, P. T., Macmillan-Lawler, M., Rupp, J. & Baker, E. K. Geomorphology of the oceans. *Marine Geology* **352**, 4-24, doi:<https://doi.org/10.1016/j.margeo.2014.01.011> (2014).

18 Joosten, H. The Global Peatland CO2 Picture: Peatland status and drainage related emissions in all countries of the world. (Wetlands International, Ede, 2010).

19 Xu, J., Morris, P. J., Liu, J. & Holden, J. PEATMAP: Refining estimates of global peatland distribution based on a meta-analysis. *CATENA* **160**, 134-140, doi:<https://doi.org/10.1016/j.catena.2017.09.010> (2018).

20 Köchy, M., Hiederer, R. & Freibauer, A. Global distribution of soil organic carbon – Part 1: Masses and frequency distributions of SOC stocks for the tropics, permafrost regions, wetlands, and the world. *SOIL* **1**, 351-365, doi:10.5194/soil-1-351-2015 (2015).

21 Lehner, B. & Döll, P. Development and validation of a global database of lakes, reservoirs and wetlands. *Journal of Hydrology* **296**, 1-22, doi:<https://doi.org/10.1016/j.jhydrol.2004.03.028> (2004).

22 Bastviken, D., Tranvik, L. J., Downing, J. A., Crill, P. M. & Enrich-Prast, A. Freshwater Methane Emissions Offset the Continental Carbon Sink. *Science* **331**, 50-50 (2011).

23 Paulsen, M. L. & Robson, B. A. in *Encyclopedia of Water* 1-12 (2019).

24 Bamber, J. L., Westaway, R. M., Marzeion, B. & Wouters, B. The land ice contribution to sea level during the satellite era. *Environmental Research Letters* **13**, 063008, doi:10.1088/1748-9326/aac2f0 (2018).

25 Schneider, A., Friedl, M. A. & Potere, D. A new map of global urban extent from MODIS satellite data. *Environmental Research Letters* **4**, 044003, doi:10.1088/1748-9326/4/4/044003 (2009).

26 Alongi, D. M. Carbon Balance in Salt Marsh and Mangrove Ecosystems: A Global Synthesis. *Journal of Marine Science and Engineering* **8**, doi:10.3390/jmse8100767 (2020).

27 Alongi, D. M. Carbon Cycling and Storage in Mangrove Forests. *Annual Review of Marine Science* **6**, 195-219, doi:10.1146/annurev-marine-010213-135020 (2014).

28 Chen, Z. L. & Lee, S. Y. Tidal Flats as a Significant Carbon Reservoir in Global Coastal Ecosystems. *Frontiers in Marine Science* **9** (2022).

29 Ingalls, A. E., Lee, C., Wakeham, S. G. & Hedges, J. I. The role of biominerals in the sinking flux and preservation of amino acids in the Southern Ocean along 170[deg]W. *Deep Sea Research Part II: Topical Studies in Oceanography* **50**, 713-738 (2003).

30 Lindberg, B. & Mienert, J. Postglacial carbonate production by cold-water corals on the Norwegian Shelf and their role in the global carbonate budget. *Geology* **33**, 537-540, doi:10.1130/G21577.1 (2005).

31 Bauer, J. E. *et al.* The changing carbon cycle of the coastal ocean. *Nature* **504**, 61-70, doi:10.1038/nature12857 (2013).

32 LaRowe, D. E. *et al.* Organic carbon and microbial activity in marine sediments on a global scale throughout the Quaternary. *Geochimica et Cosmochimica Acta* **286**, 227-247, doi:<https://doi.org/10.1016/j.gca.2020.07.017> (2020).

33 Cartapanis, O., Bianchi, D., Jaccard, S. L. & Galbraith, E. D. Global pulses of organic carbon burial in deep-sea sediments during glacial maxima. *Nat Commun* **7**, doi:10.1038/ncomms10796 (2016).

34 LaRowe, D. E. *et al.* The fate of organic carbon in marine sediments - New insights from recent data and analysis. *Earth-Science Reviews* **204**, 103146, doi:<https://doi.org/10.1016/j.earscirev.2020.103146> (2020).

35 Gorham, E. Northern peatlands: role in the carbon cycle and probable responses to climate warming. *Ecological Applications* **1**, 182 - 195 (1991).

36 Yu, Z., Loisel, J., Brosseau, D. P., Beilman, D. W. & Hunt, S. J. Global peatland dynamics since the Last Glacial Maximum. *Geophysical Research Letters* **37**, n/a-n/a, doi:10.1029/2010GL043584 (2010).

37 Rixen, T. *et al.* in *Science for the Protection of Indonesian Coastal Ecosystems (SPICE)* (eds Tim C. Jennerjahn, Tim Rixen, Hari Eko Irianto, & Joko Samiaji) 83-142 (Elsevier, 2021).

38 Regnier, P. *et al.* Anthropogenic perturbation of the carbon fluxes from land to ocean. *Nature Geosci* **6**, 597-607 (2013).

39 Cole, J. J. *et al.* Plumbing the Global Carbon Cycle: Integrating Inland Waters into the Terrestrial Carbon Budget. *Ecosystems* **10**, 171-184, doi:10.2307/27823667 (2007).

40 Saderne, V. *et al.* Accumulation of Carbonates Contributes to Coastal Vegetated Ecosystems Keeping Pace With Sea Level Rise in an Arid Region (Arabian Peninsula). *Journal of Geophysical Research: Biogeosciences* **123**, 1498-1510, doi:10.1029/2017JG004288 (2018).

41 Mazarrasa, I. *et al.* Seagrass meadows as a globally significant carbonate reservoir. *Biogeosciences* **12**, 4993-5003, doi:10.5194/bg-12-4993-2015 (2015).

42 Eyre, B. D. *et al.* Coral reefs will transition to net dissolving before end of century. *Science* **359**, 908 (2018).

43 Iglesias-Rodriguez, M. D. *et al.* Progress made in study of ocean's calcium carbonate budget. *EOS Transactions, American Geophysical Union* **83**, 365 - 375 (2002).

44 Smith, S. V. & Mackenzie, F. T. The Role of CaCO3 Reactions in the Contemporary Oceanic CO2 Cycle. *Aquatic Geochemistry* **22**, 153-175, doi:10.1007/s10498-015-9282-y (2016).

45 Hayes, C. T. *et al.* Global Ocean Sediment Composition and Burial Flux in the Deep Sea. *Global Biogeochemical Cycles* **35**, e2020GB006769, doi:<https://doi.org/10.1029/2020GB006769> (2021).

46 Middelburg, J. J., Soetaert, K. & Hagens, M. Ocean Alkalinity, Buffering and Biogeochemical Processes. *Reviews of Geophysics* **58**, e2019RG000681, doi:10.1029/2019RG000681 (2020).

1. with an addition of 0.6 10^12^ m^2^ of sandy lagoons [↑](#footnote-ref-1)
2. Open shelves = Shelves (see Table S1) - Seagrass – Macroalgae - Coral reefs (warm water) – (Coral reefs (cold water)/2) - Estuaries [↑](#footnote-ref-2)
3. Open ocean = Ocean (see Table S1) –Shelves (see Table S1); including half of the cold water reefs [↑](#footnote-ref-3)
4. Inland mineral soils = Soils (see Table S1) - Salt marshes – Mangroves - Tidal flats - Peat (high latitudes) - Peat (tropics) [↑](#footnote-ref-4)
5. Marine coastal ecosystems = Salt marshes + Mangroves + Seagrass + Macroalgae + Tidal flats + Coral reefs (warm water) + Estuaries + Open shelves [↑](#footnote-ref-5)
6. Open ocean = Open ocean (10), including half of the cold water reefs [↑](#footnote-ref-6)
7. Land = Peat (high latitudes) + Peat (tropics) + Inland mineral soils + Inland waters + Ice cover (land) + Urban areas [↑](#footnote-ref-7)
8. Total = Marine coastal ecosystems + Open ocean + Land; It represents the total surface area of the Earth which considering an Earth radius of 6371 km amounts to 510.06 10^12^ m^2^ [↑](#footnote-ref-8)
9. ((Area-min. (see Tab. S2) * POC burial-min) + (Area-max (see Tab. S2) * POC burial-max))/2 [↑](#footnote-ref-9)
10. ((Area-max. (see Tab. S2) * POC burial-max) - (Area-min (see Tab. S2) * POC burial-min))/2 [↑](#footnote-ref-10)
11. based on PIC burial rates and the organic carbon content of reef carbonates of 0.02 – 0.11 % [↑](#footnote-ref-11)
12. this estimate assumes that cold-water reef carbon burial rates are equal to 4%– 12% of those from tropical reefs. [↑](#footnote-ref-12)
13. extrapolated to global scale [↑](#footnote-ref-13)
14. this estimate assumes that cold-water reef carbon burial rates are equal 4%– 12% of those from tropical reefs. [↑](#footnote-ref-14)
15. the burial of concrete and concrete derived carbonates have been excluded. [↑](#footnote-ref-15)
